# Diagnostic Efficacy and Safety of Gadoxetate Disodium vs Gadobenate Dimeglumine in Patients With Known or Suspected Focal Liver Lesions: Results of a Clinical Phase III Study

**DOI:** 10.1177/1178623X19827976

**Published:** 2019-02-18

**Authors:** Christoph J Zech, Carsten Schwenke, Jan Endrikat

**Affiliations:** 1 Department for Radiology and Nuclear Medicine, University Hospital Basel, Basel, Switzerland; 2 SCO: SSiS Statistical Consulting, Berlin, Germany; 3 Bayer AG, Radiology, Berlin, Germany; 4 Department of Gynecology, Obstetrics and Reproductive Medicine, University Medical School of Saarland, Homburg/Saar, Germany

**Keywords:** gadoxetate disodium, gadobenate dimeglumine, focal liver lesions, hepatocellular carcinoma (HCC)

## Abstract

**Purpose:**

The aim of this study is to evaluate the diagnostic efficacy and safety of gadoxetate disodium vs gadobenate dimeglumine in patients with known or suspected focal liver lesions.

**Methods:**

This was a prospective, multicenter, double-blind, randomized, inter-individual Phase III study. The primary target—technical efficacy—was already published. Here, secondary efficacy parameters—sensitivity and specificity—and safety in specific patient populations are presented. Patients with suspected or known focal liver lesions scheduled for contrast-enhanced liver magnetic resonance imaging (MRI) were recruited and categorized in 4 a priori specified subgroups: (1) all patients, (2) patients with liver cancer (hepatocellular carcinoma [HCC]), (3) patients with cirrhosis, and (4) patients with HCC + cirrhosis. Dual multi-detector liver computed tomography (CT) served as standard of reference.

**Results:**

A total of 295 patients were included. While the overall increase in sensitivity across all 4 patient groups was comparable for gadoxetate disodium (increase from pre- to post-contrast ranging from 6.2% to 9.9%) and gadobenate dimeglumine (ranging from −2.9% to 10.0%), significant differences were seen for some of the subgroups. There was a significantly higher increase in sensitivity for gadoxetate disodium in patients with HCC (7%) and HCC + cirrhosis (12.8%) in comparison with gadobenate dimeglumine. Specificity decreased for both agents: gadoxetate disodium by −2.8% to −6.3% and gadobenate dimeglumine by −3.3% to −8.7%. Gadoxetate showed a significantly lower loss of specificity in all subgroups. Safety was comparable in both groups.

**Conclusions:**

Gadoxetate disodium proved to be an effective liver-specific MRI contrast agent. Some distinct advantages over gadobenate dimeglumine were demonstrated in patients with HCC and patients with HCC + liver cirrhosis for sensitivity and specificity in liver lesion detection.

## Introduction

Liver cancer is an increasing global problem. In the United States, the incidence has tripled over the past 20 to 30 years and is now at 7 per 100 000 Americans.^1^ Early detection and accurate characterization of liver lesions are crucial for successful therapy and overall survival of patients. Today, non-invasive diagnostic tools, eg, ultrasound, computed tomography (CT), and magnetic resonance imaging (MRI), are used worldwide. In particular, MRI in combination with liver-specific gadolinium (Gd)-based contrast agents (GBCAs) is a valuable option as no radiation is involved. In comparison with CT or to conventional MRI with extracellular contrast agent, MRI with liver-specific gadoxetate disodium has been shown to be a promising diagnostic tool for the detection of colorectal liver metastases^2^ or hepatocellular carcinoma (HCC).^3^

Gadoxetate disodium and gadobenate dimeglumine are both liver-specific MRI GBCAs. Gadoxetate disodium is approved for the detection, localization, and characterization of focal liver lesions worldwide. Gadobenate dimeglumine is approved for liver imaging in Europe.

However, they differ in a number of clinically relevant physicochemical features: first, the concentrations of the formulations and the dosing of gadoxetate disodium and gadobenate dimeglumine are different, 0.25 vs 0.5 mol/L and 0.025 vs 0.05 mmol/kg b.w. (body weight), respectively. In addition, gadoxetate disodium is characterized by a higher r1 relaxivity at 1.5 T: 4.7 (4.5-4.9) compared with 4.0 (3.8-4.2) of gadobenate dimeglumine.^4^

Both GBCAs can be used for the vascular phase (arterial and portal-venous) of liver imaging, a feature they have in common with all unspecific extracellular GBCAs. However, 50% of the administered gadoxetate disodium dose is taken up by healthy liver cells and subject to hepatobiliary excretion,^5^ while gadobenate dimeglumine features a liver uptake and biliary excretion of 0.6% to 4%.^6^ As a consequence, the recommended image acquisition windows for post-contrast biliary phase imaging is 20 to 45 min for gadoxetate disodium^7^ and 60 to 120 min for gadobenate dimeglumine.^8^ In routine practice, sufficient enhancement starts at 10 and 20 min, respectively.^9^

While there are a number of retrospective publications which directly compare both agents in general liver imaging,^10-12^ only Tirkes et al^13^ and Dioguardi Burgio et al^14^ focus on HCC. To the best of our knowledge, this is the first prospective, multicenter randomized head-to-head comparison of both agents.

The purpose of this evaluation of secondary Phase III study parameters was to assess whether the different hepatobiliary uptake between the 2 GBCAs results in any difference in patients with HCC with and without liver cirrhosis.

## Materials and Methods

### Study design

This is an analysis of secondary efficacy and safety parameters of a prospective, multicenter, double-blind, randomized inter-individual company-sponsored Phase III study comparing gadoxetate disodium and gadobenate dimeglumine in liver imaging. This study was needed for registration of gadoxetate disodium in Europe and many non-European countries. The primary target—technical efficacy parameters—was published by Filippone et al.^15^ Central and local ethics committees approved the study.

### Study population

Patients, ⩾18 years with suspected or known focal liver lesions scheduled for contrast-enhanced liver MRI, were included. As standard of reference (SOR), patients had to have a dual phase (arterial and portal-venous) multi-detector liver CT within 4 weeks before or after the MRI study procedure.

Patients were excluded if they had received any investigational drug within 30 days prior to entering this study or if they had any contraindication to contrast-enhanced MRI (eg, creatinine clearance <30 mL/1.73 m^2^, history of severe anaphylactoid reaction to contrast agents).^15^

### Contrast media

Patients received either 0.025 mmol/kg b.w. of gadoxetate disodium (Primovist, Eovist, Bayer AG, D-51368 Leverkusen, Germany, Application number: SE/H/0429/001-002 SE) or 0.05 mmol/kg b.w. of gadobenate dimeglumine (MultiHance, Bracco Imaging SpA, Milan, Italy) at 2 mL/s as single intravenous injection followed by a 20 mL 0.9% saline chaser.

### Study procedures

Patients, eligible and willing to participate in the study, gave their written consent prior to inclusion. At baseline—defined as the period within 24 h prior to contrast injection—demographic data, medical and surgical history, and medication history were recorded.

The MRI examinations were performed on 1.5 T magnetic resonance (MR) systems with phased array coils for abdominal imaging. Prior to GBCA administration, T1-weighted gradient recalled echo (GRE) sequences, 2-dimensional (2D) and 3-dimensional (3D) acquisition with fat saturation (FS), and T2-weighted fast spin echo (FSE)/turbo spin echo (TSE) or at discretion of the centers half-Fourier single shot TSE sequences were acquired. For dynamic imaging, a 3D GRE sequence was repeated during the arterial, portal-venous, and equilibrium phases (corresponding 12-20, 40-60, and 120-150 s after contrast medium injection, respectively). For hepatobiliary phase imaging, 20 and 40 min after contrast media administration, a T1-weighted 2D GRE sequence with FS and a T2-weighted FSE/TSE or Half Fourier Acquisition Single Shot Turbo Spin Echo (HASTE) sequence were acquired as already described in detail by Filippone et al.^15^

Three independent radiologists qualified and experienced in abdominal imaging evaluated the MR images in a blinded fashion. In 3 independent sessions, the blinded readers assessed pre-contrast, combined MRI images at 20 min post injection (dynamic plus post-contrast 20 min) and combined MRI images at 40 min post injection (dynamic plus post-contrast 40 min).

An additional independent blinded reading was done for the biphasic multi-detector (MD)-CT images as the SOR.

### Target variables

The target variables of this analysis were sensitivity and specificity for the detection and localization of focal liver lesions verified by a biphasic MD-CT as SOR. The unit of evaluation was the affected liver segment. Sensitivity was defined as the number of true positive affected segments divided by the number of true positive affected segments plus the number of false negative segments. Specificity was defined as the number of true negative segments divided by the number of true negative segments plus the number of false positive affected segments.

Safety parameters, in particular adverse events (AEs), were recorded on a case report form if the patient reported symptoms in response to the investigator’s open question, “How do you feel?” The investigator also assessed whether or not the reported symptoms were plausibly related to contrast administration.

### Statistics

Descriptive statistics (n, mean, standard deviation) were calculated for quantitative variables along with t-tests where appropriate; frequency counts by category were to be given for qualitative variables along with Fisher’s exact test where appropriate. Confidence intervals (CIs) were to be given where appropriate. If not otherwise stated, these intervals are 2-sided in each case and provide 95% confidence. Missing values were not replaced in the analysis of efficacy. The sensitivities and specificities and respective 95% CIs for the total population and 3 subgroups were calculated as average across the assessments of 3 blinded readers taking into account the correlation between multiple measurements (readers and segments) within the patient using an extension of the approach by Obuchowski^16^ as described in Schwenke and Busse.^17^ Significance of differences was regarded if the 95% CIs for differences did not overlap zero.

All analyses were performed using SAS Version 9.1 or higher (SAS Institute, Inc., Cary, NC, USA).

## Results

A total of 295 patients were included in 16 centers in 6 European countries (Germany, Austria, Italy, Sweden, the United Kingdom, and France) (gadoxetate disodium, n = 146; gadobenate dimeglumine, n = 149). The demographic data of both study groups were similar (Table 1).

**Table 1.** Demographic data of study population.

	Gadoxetate disodium (N = 146)	Gadobenate dimeglumine (N = 149)	*P* value^a^
Age (mean ± SD)	59.6 ± 12.6	59.6 ± 13.1	>.9999
Height (mean ± SD)	170.0 ± 8.7	168.9 ± 8.4	.2701
Weight (mean ± SD)	74.5 ± 17.4	75.2 ± 14.4	.7066
Caucasian, n (%)	138 (94.5)	145 (97.3)	.2529
Women, n (%)	57 (39.0)	61 (40.9)	.8122
Men, n (%)	89 (61.0)	88 (59.1)	.8122

a Continuous endpoints: t-test; binary endpoints: Fisher’s exact test.

More patients in the gadoxetate disodium group showed imaging signs of diffuse liver disease (liver cirrhosis or fibrosis) compared with the gadobenate dimeglumine group, 60 (41.1%) and 41 (27.5%), respectively (Table 2). Also, both patient groups differed with respect to the primary suspected liver lesion. Fewer patients in the gadoxetate disodium group were referred for contrast-enhanced MRI (CE-MRI) because of metastases and more because of HCC compared with the gadobenate dimeglumine group. However, the overall distribution of lesion types in both groups was not statistically different (*P* = .5721) (Table 3).

**Table 2.** Referral diagnosis.

Number (%) of patients with diffuse liver disease at baseline, ie, before imaging				
	Gadoxetate disodium N = 146 (100%)	Gadobenate dimeglumine N = 149 (100%)	Overall N = 295 (100%)	*P* value^a^
Diffuse liver disease	60 (41.1%)	41 (27.5%)	101 (34.2%)	.0146
Number (%) of patients with further specification of diffuse liver disease				
	Gadoxetate disodium N = 60 (100%)	Gadobenate dimeglumine N = 41 (100%)	Overall N = 101 (100%)	*P* value^a^
Liver cirrhosis	43 (71.7%)	27 (65.9%)	70 (69.3%)	.0282
Fatty infiltration	15^a^ (25.0%)	11^b^ (26.8%)	26 (25.7%)	.4170
Diffuse fibrosis	12 (20.0%)	3 (7.3%)	15 (14.9%)	.0173
Hemosiderosis/hemochromatosis	3 (5.0%)	0 (0.0%)	3 (3.0%)	.1200
Wilson’s disease	0 (0.0%)	0 (0.0%)	0 (0.0%)	>.9999
Other	3 (5.0%)	5 (12.2%)	8 (7.9%)	.7229

a Fisher’s exact test.

b One patient each had a geographic fatty infiltration.

**Table 3.** Referral diagnosis.

Number (%) of patients by primary liver lesion at baseline, ie, before imaging			
	Gadoxetate disodium N = 146 (100%)	Gadobenate dimeglumine N = 149 (100%)	*P* value^a^
Metastasis	51 (34.9%)	63 (42.3%)	.5721
HCC	37 (25.3%)	26 (17.4%)	
Hemangioma	13 (8.9%)	14 (9.4%)	
Liver cyst	13 (8.9%)	13 (8.7%)	
FNH	6 (4.1%)	5 (3.4%)	
Adenoma	2 (1.4%)	4 (2.7%)	
Cholangiocarcinoma	2 (1.4%)	3 (2.0%)	
Regenerative nodules	2 (1.4%)	1 (0.7%)	
Focal lymphoma	1 (0.7%)	0 (0.0%)	
Abscess	0 (0.0%)	1 (0.7%)	
Hydatid cyst	1 (0.7%)	0 (0.0%)	
Not assessable	15 (10.3%)	19 (12.8%)	
Other	3 (2.1%)	0 (0.0%)	

Abbreviations: FNH, focal nodular hyperplasia; HCC, hepatocellular carcinoma.

a Fisher’s exact test to test for differences in the distribution of patients to lesion types between the 2 contrast agent groups.

Sensitivity and specificity results are shown as combined assessment of all 3 blinded readers. Gadoxetate disodium consistently increased sensitivity from pre-contrast to post-contrast imaging in all 4 patient groups by 6.2% to 9.9%, while for gadobenate dimeglumine, the range of change was −2.9% to 10.0% (Table 4).

**Table 4.** Sensitivity of correctly detected liver segment affected by focal liver lesions, combined assessments of 3 blinded readers.

	All patients			HCC			Cirrhosis			HCC + cirrhosis		
	N	Sens. (%)	95% CI	N	Sens. (%)	95% CI	N	Sens. (%)	95% CI	N	Sens. (%)	95% CI
Gadoxetate disodium (pre-contrast)	123	69.7	64.1 to 75.2	29	65.0	51.8 to 78.2	33	56.6	40.6 to 72.5	23	62.2	47.2 to 77.2
Gadobenate dimeglumine (pre-contrast)	117	73.2	67.9 to 78.5	20	75.6	65.7 to 85.4	18	62.0	45.0 to 79.0	14	73.5	60.9 to 86.2
$Diff\left(Se_{pre}^{Gadoxetate\;disodium}-Se_{pre}^{Gadobenate\;dimeglumine}\right)$		−3.6	−11.2 to 4.1		−10.6	−27.0 to 5.9		−5.4	−28.7 to 17.9		−11.3	−31.0 to 8.3
Gadoxetate disodium (20 min post-pre contrast)	113	6.2	2.7 to 9.7	27	8.3	−0.1 to 16.8	31	8.0	0.8 to 15.2	21	9.9	−0.1 to 19.9
Gadobenate dimeglumine (40 min post-pre contrast)	109	6.6	2.8 to 10.3	18	1.4	−10.1 to 12.8	18	10.0	−4.0 to 24.0	14	−2.9	−13.2 to 7.3
$Diff\left(Se_{post\;\;\left(20\;min.\right)-pre}^{Gadoxetate\;disodium}-Se_{post\;\left(40\;min.\right)-pre}^{Gadobenate\;dimeglumine}\right)$		−0.3	−0.7 to 0.0		7.0	4.9 to 9.1^a^		−2.0	−4.3 to 0.2		12.8	10.4 to 15.3^a^

Abbreviations: CI, confidence interval; *Diff*, difference; HCC, hepatocellular carcinoma. *Se*, sensitivity; Sens, sensitivity.

a Statistically significant (2-sided alpha of 5%).

Specificity decreased for both agents: gadoxetate disodium by −2.8% to −6.3% and gadobenate dimeglumine by −3.3% to −8.7% (Table 5).

**Table 5.** Specificity of correctly detected liver segment affected by focal liver lesions, combined assessments of 3 blinded readers.

	All patients			HCC			Cirrhosis			HCC + cirrhosis		
	N	Spec. (%)	95% CI	N	Spec. (%)	95% CI	N	Spec. (%)	95% CI	N	Spec. (%)	95% CI
Gadoxetate disodium (pre-contrast)	119	86.5	83.7 to 89.3	31	86.3	80.3 to 92.2	36	87.5	82.2 to 92.7	25	86.3	79.2 to 93.4
Gadobenate dimeglumine (pre-contrast)	122	84.9	81.8 to 87.9	20	81.2	75.3 to 87.1	23	81.3	74.9 to 87.8	15	79.8	73.1 to 86.6
$Diff\left(Sp_{pre}^{Gadoxetate\;disodium}-Sp_{pre}^{Gadobenate\;dimeglumine}\right)$		1.6	−2.5 to 5.8		5.1	−3.3 to 13.5		6.1	−2.2 to 14.5		6.4	−3.4 to 16.3
Gadoxetate disodium (20 min post-pre contrast)	110	−2.8	−5.5 to −0.1	29	−4.7	−11.6 to 2.3	34	−4.9	−10.8 to 1.1	23	−6.3	−14.8 to 2.3
Gadobenate dimeglumine (40 min post-pre contrast)	113	−3.3	−5.8 to −0.8	19	−8.1	−14.8 to −1.4	23	−8.7	−15.2 to −2.3	15	−8.1	−15.3 to −1.0
$Diff\left(Sp_{post\;\left(20\;min.\right)-pre}^{Gadoxetate\;disodium}-Sp_{post\;\left(40\;min.\right)-pre}^{Gadobenate\;dimeglumine}\right)$		0.5	0.3 to 0.8^a^		3.4	2.0 to 4.8^a^		3.9	2.7 to 5.0^a^		1.9	0.1 to 3.7^a^

Abbreviations: CI, confidence interval; *Diff*, difference; HCC, hepatocellular carcinoma; *Sp*, specificity; Spec, specificity.

a Statistically significant (2-sided alpha of 5%).

**Figure 1.**
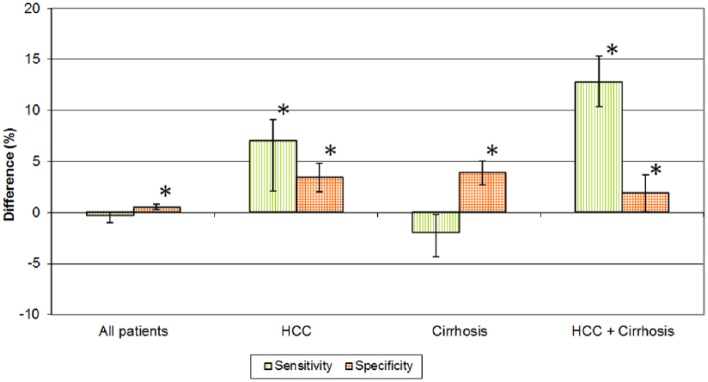
Gain or loss in sensitivity and specificity for the detection of affected liver segments for gadoxetate disodium (post [20 min]-pre) vs gadobenate dimeglumine (post [40 min]-pre), difference (%), bars indicate 95% confidence intervals. *Indicates statistical significance. HCC indicates hepatocellular carcinoma.

Considering all patients together, gain/loss of sensitivity and specificity were almost similar in both groups. Yet, there was a significantly higher increase in sensitivity for gadoxetate disodium in the subgroups of HCC and HCC + cirrhosis patients. The loss of specificity was significantly lower for all subgroups of gadoxetate disodium compared with gadobenate dimeglumine (Figure 1).

There were no deaths and no serious AEs. In each group, 9 patients (6%) experienced at least 1 AE; 9 of 12 AEs in the gadoxetate disodium group were considered by the investigator to be probably related to contrast administration, while 7 of 14 AEs in the gadobenate dimeglumine group were considered to be possibly/probably related (Table 6).

**Table 6.** Safety—patients with AEs independent of relationship to the contrast agent.

AE	Gadoxetate disodium (N = 146)	Gadobenate dimeglumine (N = 149)
Any AE	9^a^ (6%)	9^b^ (6%)
Abdominal pain	0 (0%)	2 (1%)
Accidental injury	0 (0%)	1 (1%)
Anxiety	1 (1%)	0 (0%)
Asthenia	0 (0%)	1 (1%)
Back pain	0 (0%)	1 (1%)
Chest pain	1 (1%)	0 (0%)
Chills	0 (0%)	1 (1%)
Extrasystoles	1 (1%)	0 (0%)
Increased salivation	1 (1%)	0 (0%)
Injection site reaction	0 (0%)	1 (1%)
Nausea	1 (1%)	2 (1%)
Rash	0 (0%)	1 (1%)
Rhinitis	0 (0%)	1 (1%)
Sweating increased	0 (0%)	1 (1%)
Taste perversion	1 (1%)	0 (0%)
Vasodilation	5 (3%)	1 (1%)
Vertigo	1 (1%)	0 (0%)
Any serious AE	0 (0%)	0 (0%)
Any fatal AE	0 (0%)	0 (0%)

Abbreviation: AEs, adverse events.

a Overall 12 AEs in 9 patients.

b Overall 14 AEs in 9 patients.

## Discussion

This prospective, multicenter, double-blind, randomized, inter-individual Phase III study evaluated sensitivity, specificity, and safety of gadoxetate disodium vs gadobenate dimeglumine in specific patient populations. To the best of our knowledge, this is the first head-to-head comparison of these 2 agents using this standard of study design.^18^

Both contrast agents increased the sensitivity of liver lesion detection while losing some specificity. A statistically significant higher increase in sensitivity in the subgroups of patients with HCC and HCC + cirrhosis was seen for gadoxetate disodium. Applying the 6 tier hierarchical model of diagnostic efficacy by Thornbury et al,^19^ this study would be on Tier 2, “Diagnostic—accuracy efficacy.” So far, no other prospective comparative studies comparing these 2 agents in patients with focal liver lesions have been reported. However, a number of studies on technical efficacy according to Thornbury (ie, signal intensity, contrast, image quality, delineation)^19^ focusing on liver cancer have been published.

Dioguardi Burgio et al reported a retrospective, inter-individual study in 51 patients with HCC. Capsule appearance was more frequently seen on gadobenate dimeglumine MRIs compared with gadoxetate disodium.^14^ Clinical impact of this result was not shown. Also, Tirkes et al^13^ presented retrospective, single-center, inter-individual data on 95 patients with HCC. The overall difference in contrast-to-noise-ratios did not reach statistical significance. Again, clinical aspects were not discussed.

In the light of currently available comparative study results, the findings presented here appear more meaningful for daily practice in detecting and characterizing focal liver lesions.

The safety profile of both agents was similar with respect to AEs and drug-related AEs. This is in line with large safety reviews of both agents. Endrikat et al^20^ reported 10.1% of AEs of which 4.1% were classified as related to gadoxetate disodium administration in an analysis of 12 clinical development studies, including 1989 patients. Likewise, Shellock et al^21^ found 18% of AEs with 14% AEs considered related to gadobenate dimeglumine administration in a review of 79 clinical studies, including 2982 patients. In addition, so far no case of nephrogentic systemic fibrosis (NSF) has been reported even in patients with moderate to severe renal impairment as shown by Lauenstein et al^22^ in a prospective observational multicenter study in 357 patients.

With regard to Gd presence in the brain, 3 imaging studies have been published to date with gadoxetate disodium.^23-25^ Two of these publications reported no signal increase (SI) increase after up to 15 or 18 gadoxetate disodium administrations.^23,24^ The third study by Kahn et al^25^ reported an SI increase in the dentate nucleus (DN) of patients who received a number of injections ranging from 11 to 37 while no SI was seen in patients with less than 10 gadoxetate disodium injections. The finding that an increased SI becomes visible for gadoxetate disodium only after a distinctly higher number of administrations is not unexpected given the dose dependency of the SI increase and the fact that gadoxetate disodium is administered only at a quarter of the Gd dose (0.025 mmol Gd/kg b.w.) of multi-purpose GBCAs. In addition, this contrast’s unique dual elimination pathway (50% renal, 50% hepatobiliary), and higher stability than all other linear GBCAs may also contribute to a lower systemic Gd burden. In 2013, Davenport et al presented a phenomenon called “acute transient dyspnea” (aka as “breathing artifacts”) occurring significantly more often with gadoxetate disodium compared with gadobenate dimeglumine. This was alleged to be deleterious for dynamic/arterial phase image quality, but patients did not require treatment.^26^ Although this Phase III study was meticulously monitored, these effects were not seen here, neither clinically nor during image evaluation. As of today, this topic is still in scientific debate.^27-29^

Some limitations need to be addressed: (1) although treatment allocation was randomized, the treatment groups were somewhat different with respect to prevalence of liver cirrhosis and diffuse fibrosis. This might have had an impact on pre-treatment sensitivity and specificity. To what degree the different baseline MRIs influenced the pre-post contrast comparison is unknown. (2) This evaluation focused on secondary efficacy parameters. Therefore, all statistical testing was done descriptively. (3) The SOR was CT imaging, not histopathology. Nowadays, we may assume that MRI is superior to CT for detecting HCC nodules and liver metastases. However, because the main focus of this study was to compare 2 MRI contrast agents, we may have a potential bias in the absolute values (as compared with other studies), while being confident that the differences of the 2 MRI contrast agents in diagnostic performance can still be interpreted. (4) No dedicated lesion tracking and no evaluation based on lesion type or size was performed. (5) No dedicated evaluation was carried out about the specific impact of the 3 imaging phases (arterial, venous, or hepatobiliary phase) on the results.

In conclusion, gadoxetate disodium proved to be an effective liver-specific MRI contrast agent. Distinct advantages over gadobenate dimeglumine were demonstrated in patients with HCC and patients with HCC + liver cirrhosis for sensitivity and specificity in liver lesion detection.
